# Effect of three kinds of natural preservative cocktails on vacuum‐packed chilled pork

**DOI:** 10.1002/fsn3.1535

**Published:** 2020-05-20

**Authors:** Chen Li, Yuhang Han, Sanhong Fan, Lizhen Ma, Yi Zhang, Benjamin Kofi Simpson

**Affiliations:** ^1^ School of Life Science Shanxi University Taiyuan China; ^2^ Department of Food Science Tianjin Agricultural University Tianjin China; ^3^ Department of Food Science and Agricultural Chemistry McGill University (Macdonald Campus) Ste‐Anne‐de‐Bellevue QC Canada

**Keywords:** biogenic amines, chilled pork, microbial profiles, natural preservative cocktails

## Abstract

The aim of this study was to investigate the effects of three different natural preservatives on the microbial profile, the total volatile base nitrogen (TVB‐N), and biogenic amine contents of vacuum‐packed chilled pork during storage at 4°C. Solution A comprised of tea polyphenols, chitosan, spice extract, propolis, and nisin. Solution B comprised of clove extract, cassia bark extract, ginger juice, garlic juice, and *lactobacillus* fermentation solution. Solution C consisted of only *lactobacillus* fermentation solution. The results indicated that solution A was a good natural preservative with higher bacteria inhibitory effect and higher sensory score than B and C. Besides the effect on appealing color, solution B could inhibit microbial activity although its inhibition effect was not as good as solution A. Thus, solution A could be used as a good preservative in industry. Solution C could inhibit the initial growth of *Pseudomonas* and partially inhibited the growth of *Enterobacteriaceae*; however, the content of putrescine in the pork treated with solution C was as high as 30.14 ± 2.89 mg/kg after 21 days of storage at 4°C. Hence, solution C is not an ideal preservative for vacuum‐packed chilled pork.

## Graphical abstract

The effects of three kinds of preservation solutions on chilled pork were determined. Microbial analysis, TVB‐N, and sensory parameters were determined during the storage. Solution A is a good natural preservation solution on vacuum‐packed chilled pork.

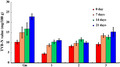

## 1 INTRODUCTION

Food preservation continues to be of major public health concern to consumers, food scientists, and food technologist, as well as government regulatory agencies. It is reported that about one‐third of food produced globally for human consumption is lost along the food supply chain due to spoilage and putrefaction each year (Kiran, Trzcinski, Ng, & Liu, 2014). Especially, the preservation and control of meat spoilage have been an issue attracting socioeconomic attention for both food safety and food security reasons. Since chilled meat is preferred to frozen meat due to its higher nutritive quality, it has a growing market share and has become mainstream of raw meat consumption (Ngapo, Riendeau, Laberge, & Fortin, 2012; Ngapo, Riendeau, Laberge, Leblanc, & Fortin, 2012). Thus, the chilled meat technologies play a key role in meat processing industry (Chang et al., 2019). However, owing to its nutrient‐dense nature as well as its high moisture content, chilled meat is comparatively more susceptible to growth and proliferation of psychrophile bacteria versus frozen meat during the production, handling, and sales. This results in problems such as difficulty of market circulation, shorter shelf‐life, and safety concerns (Casaburi, Piombino, Nychas, Villani, & Ercolini, 2015; Jiang et al., 2010). Therefore, how to prolong the preservation time and useful shelf‐life of chilled meat becomes an urgent topic.

In recent years, techniques to keep the quality of chilled pork during storage have been widely studied. There are a variety of options to extend the shelf‐life of meat, such as microbial decontamination, smoking or radiation treatments (Kanatt, Chander, & Sharma, 2005; Kim et al., 2014), vacuum ( Blixt & Borch, 2002) or modified atmosphere packaging (Fraqueza & Barreto, 2009; Pérez‐Rodríguez, Zamorano, Posada‐Izquierdo, & García‐Gimeno, 2014), and low temperature storage. As reported, combined preservation techniques based on the hurdle concept could achieve better effects by posing additional barriers to compensate for the shortcomings of different methods applied individually (Salcedo‐Sandoval, Cofrades, Ruiz‐Capillas, Carballo, & Jiménez‐Colmenero, 2015; Xu, Huang, Huang, Xu, & Zhou, 2012). Furthermore, considering consumer aversion to the use of chemicals and antibiotics as food processing aids, the use of natural preservative compounds and their cocktails is of paramount interest among researchers.

The commonly used natural disinfectant and preservation solutions include green tea polyphenols (Bañón, Díaz, Rodríguez, Garrido, & Price, 2007; Perumalla & Hettiarachchy, 2011); chitosan (Rao, Chander, & Sharma, 2005); propolis, nisin, and lysozyme (Kijowski & Leisnierowski, 1995); rosemary and clove (Angiolillo, Conte, & Nobile, 2014); cassia bark and fresh ginger (Zhang, Kong, Xiong, & Sun, 2009); and garlic (Shah, Bosco, & Mir, 2014). Thus far, single‐component natural disinfectant and preservation solutions have been studied with respect to their capacity to extend the useful shelf‐life of chilled meat. However, only very few studies have thus far been reported on the effects of combination treatments using natural cocktails comprised of several antimicrobial and preservative components.

The aim of this study was to investigate the preservation ability of three kinds of natural antimicrobial and preservative cocktails and evaluate their effect on the prolongation of chilled pork shelf‐life based on two important indexes including microbiologic analysis and physicochemical changes.

## 2 MATERIALS AND METHODS

### 2.1 Chemicals and solutions

All microbiological media were from Shanghai Sangon Biotech Co., Ltd. All other chemicals were from Beijing Solarbio Science & Technology Co., Ltd., unless otherwise indicated.

Green tea polyphenol, chitosan, nisin, clove, cassia bark, and rosemary were purchased from Linyi Lumeng Food Co., Ltd. Aqueous extract of propolis was purchased from Shaanxi Laofengnong Biotechnology Co., Ltd. Fresh ginger and garlic were purchased from local supermarket in Taiyuan City. A strain of lactic acid bacteria (LAB) which could produce bacteriocin was provided by Biology Institute of Shanxi.

The spice extract was prepared as follows. A volume of 300 ml water was added to 30 g spice, for instance rosemary, clove, and cassia bark, respectively. After heating to boiling and holding for 30 min, the mixture was filtrated. The volume of filtrate was made to 300 ml with sterile water and defined as spice extract, which was rosemary extract, clove extract, and cassia bark extract, respectively.

Ginger juice and garlic were prepared by homogenizing 10 g fresh ginger and garlic with 100 ml sterile water, respectively.

Lactic acid bacteria‐fermented solution was prepared by incubating lactic acid bacteria with deMan Rogosa Sharpe (MRS) liquid medium to a log CFU/ml of 5.5.

### 2.2 Raw materials

Chilled pork samples (dorsal muscle) were obtained from a local supermarket in Taiyuan City, China. The tendons and velums were removed. Then, the separated pork was cut into small pieces of 100 g around under sterile conditions in a Clean Bench (SW‐CJ‐1FD, Shanghai Boxun Industry ＆ Commerce Co., Ltd.).

### 2.3 Experimental design

Pork blocks were randomly separated into four groups in the experiment, and there were 30 pork blocks in each group. The pork blocks soaked in sterile distilled water, and preservation solutions A, B, and C were defined as control group and experimental groups 1, 2, and 3, respectively. After soaked for 1 min, the pork blocks were drained for 2 ～ 5 min until water dripping ceased. Then, the pork blocks were vacuum packaged using a vacuum packing machine (Jinan Xunjie Packing Machinery Co., Ltd.) and kept in refrigerator at 4°C. The vacuum packaging tube was made of nylon/polyethylene with an oxygen permeating rate of 90 ml m^−1^ 24 hr^−1^ atm^−1^, under 23°C and 60% relative humidity. Analyses were performed for bacterial count, total volatile basic nitrogen (TVB‐N) value, and biogenic amines at 0, 7, 14, and 21 days (starting 3 hr after they were put into the refrigerator). Preservation solution A was consisted of green tea polyphenol (5 g/L), chitosan (5 g/L), rosemary extract (25 ml/L), propolis (1 g/L), and nisin (15 g/L). Preservation solution B was consisted of clove extract (132.5 ml/L), cassia bark extract (193.5 ml/L), *lactobacillus* fermentation solution (176.5 ml/L), ginger juice (91.6 ml/L), and garlic juice (61.5 ml/L). Preservation solution C was *lactobacillus* fermentation solution (Leroi, Arbey, Joffraud, & Chevalier, 1996; Verma, Banerjee, Dwivedi, & Juneja, 2014).

### 2.4 Microbial analysis

Total bacterial count was measured according to China national food safety standard GB4789.2‐2016 and expressed in Log_10_CFU/g (CFU, colony‐forming unit) (Liu et al., 2019). The detailed procedure was as follows: Samples (25 g) were taken aseptically from the pork blocks and cut up with steam sterilized scissors. The cut chips were suspended in 225 ml sterile normal saline and agitated for 30 min in a shaker (HYG‐A, Taicang experimental equipment factor, China). An aliquot of 1 ml supernatant as well as two subsequent decimal dilutions was spread on the surface of agar medium. Numbers were determined on plate count agar (PCA) for total microbe counts at 30°C for 48 hr, streptomycin thallous acetate actidione (STAA) agar for *Brochothrix thermosphacta* at 25°C for 48 hr, violet red bile glucose agar (VRBG) for *Enterobacteriaceae* at 35°C for 24 hr, deMan Rogosa Sharpe Agar (MRSA) for lactic acid bacteria at 35°C for 48 hr, Pseudomonas CN agar for *Pseudomonas* at 25°C for 48 hr, and PCA for Psychrophile at 7°C for 10 days.

After incubation of inoculated Petri dishes at appropriate temperatures, the counts of characteristic colonies were enumerated, calculated, and displayed in CFU.

### 2.5 TVB‐N value

Total volatile basic nitrogen (TVB‐N) value was measured according to China national food safety standards GB/T5009.228‐2016, following the half minimal diffusion method (Liu et al., 2019). Briefly, a total of 1 ml of H_3_BO_3_ (20 g/L) solution and a drop of mixed indicator (methyl red ethanol solution and bromocresol green ethanol solution in a volume ration of 1:5) were added into the inner chamber of diffusion vessel. An aliquot of 1 ml of sample filtrate and 1 ml of saturated K_2_CO_3_ solution was added into the outer chamber of the diffusion vessel and mixed thoroughly. The reaction solution was titrated with 0.01 mol/L HCl reagent after 2 hr at 37°C. The amount of TVB‐N was calculated using the equation below, where v_1_ and v_2_ are the titration volumes of the test sample (ml) and blank (ml), respectively; c is the concentration of HCl (moL/L), and m is the weight of pork sample (g) (Pan et al., 2018).

$$\text{TVB}-N\;\left(\text{mg}/100\;g\right)=\frac{(v1-v2)\times c\times 14}{m\times 1/100}\times 100$$

### 2.6 Determination of biogenic amines

Lean meat was selected from the pork blocks, soaked in 0.4 M perchloride acid, and homogenized in a mixer (Sorvall Omni) for 2 min at 9,000 *g*. Biogenic amines were extracted and centrifugated at 6,800 *g* for 10 min at 4°C. The supernatant was derivatized with dansyl chloride, and the contents of tyramine, putrescine, cadaverine, histamine, spermine, tryptamine, and spermidine were determined using an HPLC instrument (Shimadzu LC‐10A) on a C18 reverse‐phase column, following the method described by Latorre‐Moratalla et al., with some modifications (Latorre‐Moratalla et al., 2009). The detection condition was as follows: Excitation and detection wavelength were 340 nm and 440 nm, respectively; elution rate was 1.0 ml/min, and derivation solution flow rate was 0.5 ml/min.

### 2.7 Color measurement

The chilled pork samples from each treatment were subjected to color measurement using a TC‐P2 full‐automatic color difference meter (Beijing Xinaoyike Photoelectric Technology Co., Ltd). Before measurement, the colorimeter was calibrated with a white tile. Color coordinates including *L* ^*^ (lightness), *a* ^*^ (redness/greenness), and *b* ^*^ (yellowness/blueness) were measured. Three measurements were performed on each sample.

### 2.8 Sensory analysis

Sensory analysis including aroma, flavor, juiciness, tenderness, and color of chilled pork was carried out by the methods for sensory evaluation of meat (Bradley et al., 2011; Brenesselova et al., 2015). Sensory examination was performed by six trained evaluators on day 21 of storage. The chilled pork samples were examined by panelists for color and elasticity. After the samples were boiled for 30 min, every panelist examined the chilled meat for the indexes of aroma, flavor, juiciness, and tenderness. Each sample was coded with a random letter. The descriptors were measured using a 5‐point intensity line scale for each index; for instance, number 5 indicated the highest quality of evaluated parameter and the number 0 indicated the lowest quality.

### 2.9 Data analyses

Samples were analyzed in triplicate, and the results were shown as mean ± standard deviation. All the experimental data were analyzed with SPSS software (8.0 edition). One‐way analysis of variance was used to evaluate statistical significance of differences between four groups of meat with different treatments. The level of significance was *p* < .05.

## 3 RESULTS

### 3.1 Microbial analyses

**FIGURE 1:**
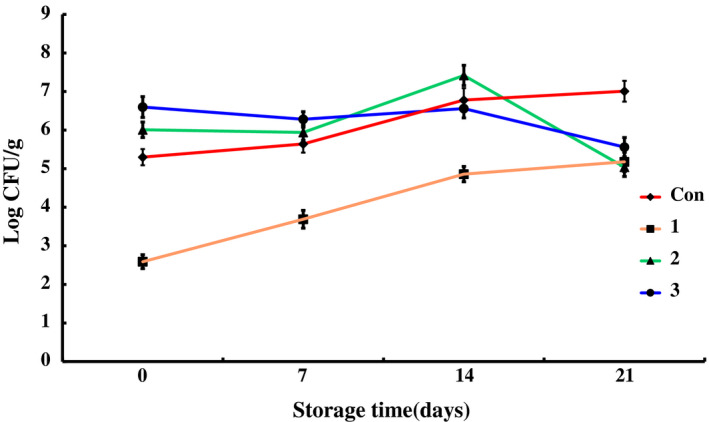
The changes in the total microbe counts of chilled pork treated with natural preservation solution and stored at 4°C

**FIGURE 2:**
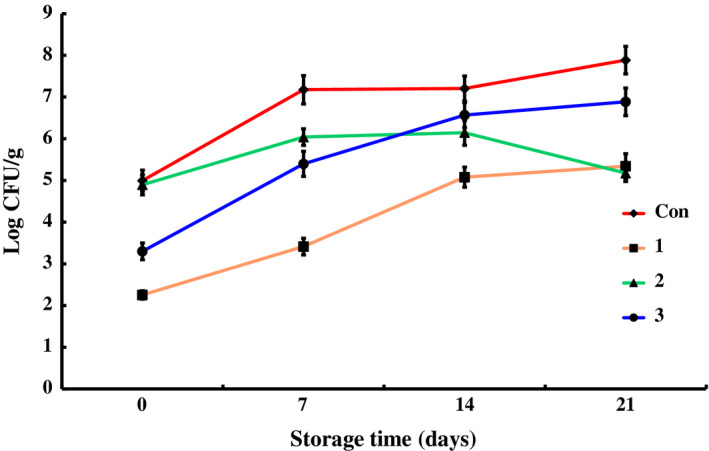
The changes in the *Pseudomonas* of chilled pork treated with natural preservation solution and stored at 4°C

**FIGURE 3:**
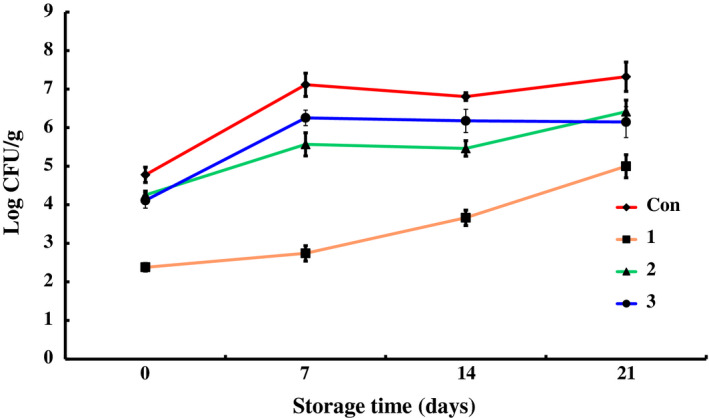
The changes in the *Enterobacteriaceae* of chilled pork treated with natural preservation solution and stored at 4°C

**FIGURE 4:**
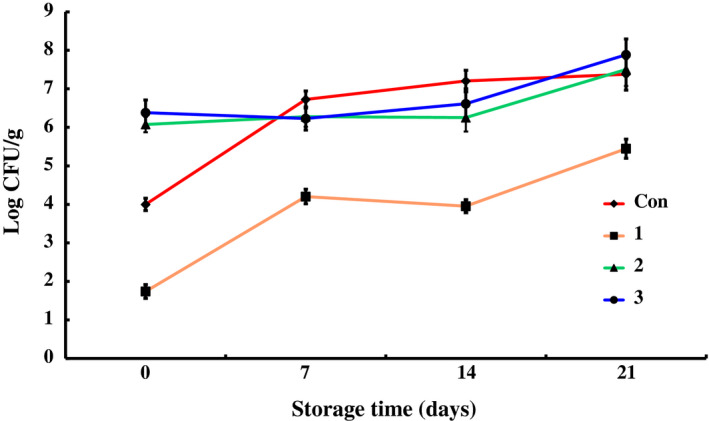
The changes in the Lactic acid bacteria of chilled pork treated with natural preservation solution and stored at 4°C

**FIGURE 5:**
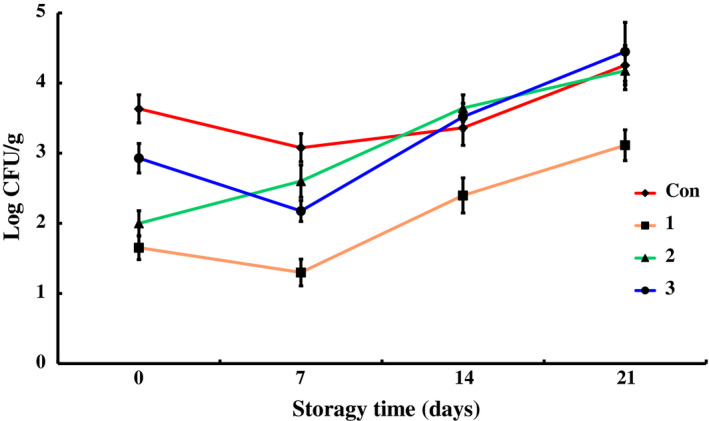
The changes in the *Brochothrix thermosphacta* of chilled pork treated with natural preservation solution and stored at 4°C

**FIGURE 6:**
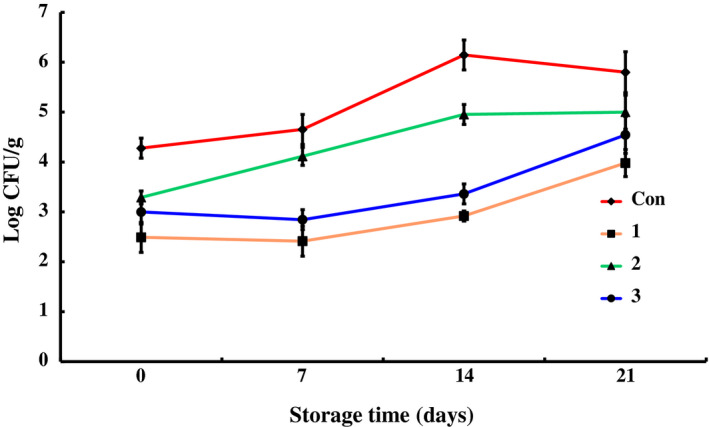
The changes in the psychrophile of chilled pork treated with natural preservation solution and stored at 4°C

The data for counts of total bacteria, *Pseudomonas*,* Enterobacteriaceae*, total lactic acid bacteria, *Brochothrix thermosphacta*, and psychrophiles in different groups during the storage at 4 ± 1°C are shown in Figures 1, 2, 3, 4, 5, 6.

Total bacterial counts (TBC) of control samples increased during the storage. It reached to 7.01 ± 0.27 log CFU/g on day 21. Similar results were recorded in the work of Zhao et al (Zhao et al., 2015), who stored vacuum‐packed chilled pork at 0°C. In their work, the TBC was 6.50 ± 0.34 log CFU/g on day 21. The total microbe counts decreased by 2 log CFU/g on the surface of the pork treated with preservation solution A stored at 4°C for 3 hr (Figure 1). The TBC in group 1 varied slowly during the storage period and increased to 5.18 ± 0.25 log CFU/g on day 21. Significant difference of TBC between group 1 and control group was observed (*p* < .01). Before the 14th day, the TBCs in groups 2 and 3 were a little higher than that of control group, but no significant difference was obtained（*p* > .05). However, after the 14th day, the TBCs in groups 2 and 3 were much lower than that of control group（*p* < .05). The values in groups 2 and 3 were 5.04 ± 0.24 and 5.56 ± 0.25 log CFU/g, respectively, on the 21st day. The reason for the above phenomenon is that lactic acid bacteria in preservation solutions B and C were active, resulting in a higher total microbial count in groups 2 and 3 from the dominant bacteria colony of lactic acid bacteria at the initial storage (Pipek et al., 2006). But due to the competitive advantage of lactic acid bacteria, the propagation of other bacteria was inhibited, resulting in a lower total microbial count than that of control group at the end of the storage period. As shown in Figure 1, all the three preservation cocktails could obviously decrease the microbial counts on the chilled pork. Especially, solution A was markedly effective during the whole storage of the chilled pork, while solutions B and C only exhibited effectiveness toward the end of the storage by competitive inhibition.

Preservation cocktail A could effectively inhibit *Pseudomonas* growth, as shown in Figure 2. Comparing to control group, the initial number of *Pseudomonas* was about 3 log CFU/g lower and the growth of *Pseudomonas* during the whole storage period was quite slower in group 1. The value of *Pseudomonas* counts was 5.34 ± 0.30 log CFU/g on the 21st day. This value was significantly lower in comparison with the control group (*p* < .01). Preservation C also showed inhibitory effect on *Pseudomonas* with 2 log CFU/g decrease of initial counts. But during storage, the counts increased rapidly up to 6.89 ± 0.33 log CFU/g on the 21st day. Though preservation B did not exhibit obvious inhibitory effect at the beginning, the *Pseudomonas* in group 2 increased quite slowly during the whole storage and the counts was similar with group 1 on the 21st day, which was obviously less than the value of control group and group 3. The *Pseudomonas* in control group increased slowly and continuously, and reached to 7.89 ± 0.30 log CFU/g on the 21st day. Thus, all the three preservation solutions showed inhibitory effects on *Pseudomonas*: A was effective during the whole period, while B was effective at the late stage and C at the early stage. *Pseudomonas* is an aerobic microbe. Thus, under vacuum packing condition and effect of preservation, it increased slowly. However, in the control group, the value of *Pseudomonas* exceeded 7.00 log CFU/g on the 21st day, implying that chilled pork began to decay. There were at least two conditions responsible for the decay of control group. One reason was that no preservation solution was used in control group, and the other one was related to the oxygen permeability of the package bag. The O_2_ permeation rate of the tube used in the experiment was 2 ml m^−2^ h^−1^ atm^−1^ at 23°C, while each *Pseudomonas* cell just needs 9.7 × 10^–8^ μl oxygen per day for growth and propagation (Slabbert & Grabow, 1986). Therefore, 480 ml oxygen permeated each day could amply meet the requirements of 5 × 10^8^ cfu/ml *Pseudomonas*. At the initial stage, respiration action of the pork would exhaust most of them. With time, the pork stopped respiration and the oxygen permeated was mainly used by *Pseudomonas* which resulted in the counts increase in the control group at late stage.

As shown in Figure 3, preservation solution A exhibited obvious inhibitory effect on *Enterobacteriaceae*. The number of group 1 was more than 2 log CFU/g lower than that of control group at the initial stage and increased slowly during the whole storage period. On the 21st day, the log CFU/g value of group 1 was 5.0 ± 0.3 log CFU/g, which was lower than and significantly different (*p* < .01) from the value of control group. Preservation solutions B and C also showed some inhibitory effects on *Enterobacteriaceae*, but much weaker than A. On the 21st day, the log CFU/g values of groups 2 and 3 were 6.415 ± 0.31 log CFU/g and 6.146 ± 0.40 log CFU/g, respectively, which were much lower than that of control group (*p* < .05). These results indicate that preservation solution A had stronger inhibitory effect on *Enterobacteriaceae* than B and C.

As shown in Figure 4, the counts of lactic acid bacteria of groups 2 and 3 on the 1st day were up to 2 log CFU/g higher than that of control group due to the presence of active bacteria. However, the counts did not increase further until the 14th day, and the values reached to 7.00 Log CFU/g on the 21st day, which was much higher than that of control group. Preservation solution A showed significant inhibitory effect on lactic acid bacteria in chilled pork with the initial counts 2 log CFU/g lower than that of control group (*p* < .01). This finding is consistent with the report by Jiang *et al*. investigated the bacterial communities of vacuum‐packed chilled pork during storage using PCR‐denaturing gradient gel electrophoresis (DGGE), who found that the composition of the microbes varied at different stages of storage, and *Lactobacillus* was the dominant species at the end of storage (Jiang et al., 2010).

Preservation solutions A, B, and C could all inhibit the initial growth of *Brochothrix thermosphacta* in chilled pork as shown in Figure 5. The initial inhibitory effect of preservation solutions A and B on *Brochothrix thermosphacta* was significant, while C was insignificant. During the whole storage period, the counts increased slowly in group 1 and remained lower than that of the control group. However, the log CFU/g values of groups 2 and 3 increased rapidly and reached to the same level with the control group from day 7. Compared with the total microbe counts and *Pseudomonas* counts, the number of *Brochothrix thermosphacta* in all experimental groups increased more slightly and the values were only 3.11 ± 0.22 log CFU/g in group 1 and around 4.0 log CFU/g in the other three groups by day 21, which implied *Brochothrix thermosphacta* was not the predominant bacteria in the chilled pork.

The change in psychrophiles in chilled pork treated with preservation solutions was presented in Figure 6. The initial log CFU/g values of all treated samples were lower than that of the control group. This tendency lasted from day 1 to day 21, and the log CFU/g values of psychrophiles in groups 1, 2, and 3 were 3.98 ± 0.27, 5.00 ± 0.34, and 4.54 ± 0.37 log CFU/g, respectively, which were lower than the 5.80 ± 0.41 log CFU/g of the control group (*p* < .05).

### 3.2 Physical and chemical indexes

**FIGURE 7:**
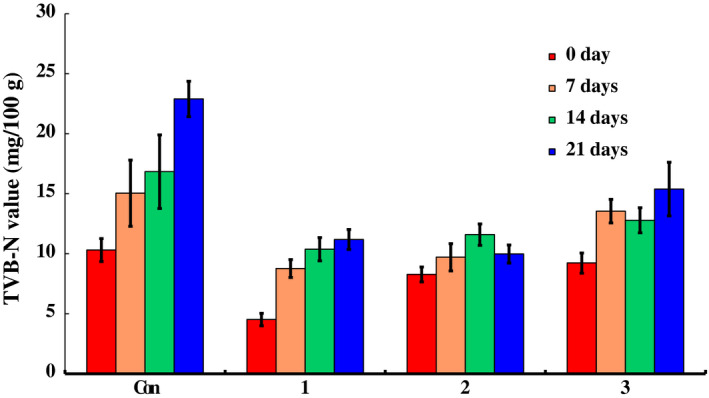
The changes in the TVB‐N value of chilled pork treated with natural preservation solution and stored at 4°C

Total volatile basic nitrogen (TVB‐N) content is an important reference index for evaluating pork freshness (Huang, Zhao, Chen, & Zhang, 2014). The experimental results of TVB‐N value and biogenic amines of all groups stored at 4 ± 1°C are shown in Figure 7 and Table 1, respectively.

**Table 1:** Biogenic amine content and color parameter for vacuum‐packed chilled pork on day 21 at 4°C

Sample indexes	Control group （mg/kg）	Group 1 （mg/kg）	Group 2 （mg/kg）	Group 3 （mg/kg）
Tyramine	ND	0.16 ± 0.02^b^	2.20 ± 0.15^a^	ND
Putrescine	1.14 ± 0.05^b^	1.03 ± 0.05^c^	1.02 ± 0.04^c^	30.14 ± 2.89^a^
Cadaverine	4.36 ± 0.33^a^	4.33 ± 0.30^a^	4.06 ± 0.27^a^	3.67 ± 0.45^b^
Histamine	ND	ND	ND	ND
Spermine	2.24 ± 0.26^b^	1.42 ± 0.04^c^	2.09 ± 0.20^b^	3.79 ± 0.18^a^
Tryptamine	2.63 ± 0.38^a^	1.20 ± 0.04^b^	1.51 ± 0.03^b^	ND
Spermidine	17.75 ± 1.61^b^	21.33 ± 3.06^a^	20.21 ± 2.56^a^	3.01 ± 0.09^c^
*L* ^*^	36.79 ± 1.41^a^	40.93 ± 3.39^a^	46.49 ± 3.01^b^	45.99 ± 3.77^b^
*a* ^*^	10.54 ± 1.14^a^	11.10 ± 1.58^a^	11.98 ± 1.82^a^	9.84 ± 1.73^a^
*b* ^*^	13.37 ± 1.05^a^	14.55 ± 1.7^a^	15.72 ± 2.24^a^	12.50 ± 0.51^a^

Note

Means in the lines with the same superscript (a, b, c) do not differ significantly.

As indicated in Figure 7, the TVB‐N values of control group were consistently higher than those of the groups treated with the preservation solutions throughout the storage period. The TVB‐N level of the control group reached 22.90 ± 1.47 mg/100 g by day 21. Clearly, the TVB‐N levels of all the test groups were lower than 15.00 mg/100 g with the level of group 1 being the lowest at 11.19 ± 0.83 mg/100 g. These results indicate that all the preservation solutions achieved higher freshness retention of chilled pork, and in this regard, preservation solutions A and B were more effective than solution C. These TVB‐N analyses results were consistent with the total microbe counts data. For example, when the total microbe counts of the control group reached to the highest value (7.01 ± 0.27 log CFU/g), the TVB‐N value attained the highest value (22.90 ± 1.47 mg/100 g) on day 21 which was mainly due to accelerated protein degradation and accumulated biogenic amines by a large number of bacteria (Liu et al., 2018).

As indicated in Table 1, the content of the biogenic amines in groups 1, 2, and control group was relatively low except spermidine. For example, histamine even could not be detected. The contents of putrescine, spermine, and tryptamine in group 1 were significantly lower than that of the control group (*p* < .05), while the contents of cadaverine and tyramine were also lower but not significant (*p* > .05). As to group 3, except putrescine (30.14 ± 2.89 mg/kg), the contents of other biogenic amines were relatively low. The content of spermidine in group 3 was 6 times less than that of group control. Hitherto, neither tyramine nor tryptamine was detected. As previously reported, putrescine and cadaverine were released during the decomposition of amino acid, and putrescine was produced mainly due to protein degradation by *Pseudomonas* (Krizek, Smith, & Phebus, 1995). The content of putrescine in group 3 reached a high value of 30.14 ± 2.89 mg/kg. This high value may be related with *Pseudomonas* considering the high value of *Pseudomonas* in group 3 (6.886 ± 0.33 log CFU/g) at the end of storage. Another reason for the high content of putrescine in group 3 may be related with lactic acid bacteria. The pork blocks in group 3 were soaked with lactic acid bacteria fermentation solution, and the counts of lactic acid bacteria reached the highest on day 21 of storage. Biogenic amines could be accumulated from reactions catalyzed by amino acid decarboxylase of lactic acid bacteria (Poveda, Ruiz, Sesena, & Palop, 2017).

Meat color measurements showed that the *L* ^*^ values in groups 2 and 3 were significantly higher than that in groups control and 1 (*p* < .05), while *a* ^*^ and *b* ^*^ did not show significant changes in the four groups (*p* > .05) (Table 1).

### 3.3 Sensory indexes

The sensory evaluation of the samples on day 21 is presented in Table 2. The color of chilled pork in groups 1, 3, and the control was purple. This was the normal color of vacuum‐packaged chilled pork and indicated that the muscle myoglobin existed mainly in reduced form (Hood & Riordan, 1973; Millar, Moss, & Stevenson, 1996). The color of pork is of major concern to the pork industry, and the optimum surface color of fresh pork should be reddish‐pink (Mancini & Hunt, 2005). Among the 4 groups, the color of chilled pork in group 2 was a more attractive bright‐red color with a highest lightness value (Table 2) on day 21, and it remained stable throughout the entire storage period.

**Table 2:** Results of sensory analysis of vacuum‐packed chilled pork on day 21 at 4°C

Treatment	Control	1	2	3
Color	3.8 ± 0.65	3.8 ± 0.78^a^	4.2 ± 0.92^a^	3.6 ± 0.39^a^
Elasticity	3.5 ± 0.43	4.2 ± 0.55^a^	4.0 ± 0.69^a^	4.8 ± 0.40^a^
Flavor	–	4.4 ± 0.36^b^	3.8 ± 0.44^a^	3.4 ± 0.59^a^
Aroma	–	4.2 ± 0.47^a^	3.6 ± 0.64^a^	3.6 ± 0.42^a^
Juiciness	–	3.8 ± 0.39^a^	3.4 ± 0.73^a^	3.2 ± 0.68^a^
Tenderness	–	4.2 ± 0.56^a^	3.6 ± 0.35^a^	3.8 ± 0.21^a^
Sum score	–	24.6 ± 3.12^a^	22.6 ± 3.77^a^	21.4 ± 2.69^a^

Note

Means in the lines with the same superscript (a, b) do not differ significantly.

The texture of meat is also an important factor in the consumer's choice. On the day 21 of storage, chilled pork treated with solution A showed the best elasticity among 4 groups. The meat elasticity of control group decreased obviously. Both groups 2 and 3 improved the meat elasticity comparing with control group. Over a storage of 21 days, the sample of control group was decayed with unacceptable odor. However, the odor of three groups treated with natural preservatives remained acceptable for a period of 21 days under vacuum‐packed condition at 4°C. After cooking, the flavor, aroma, juiciness, and tenderness of the three groups were evaluated. Samples in group 1 received the highest sensory scores, subsequently group 2 and group 3. Except the color score of group 1 was less than that group 2, samples in group 1 achieved higher scores in all other taste items comparing to samples of groups 2 and 3. The results of sensory evaluation were in agreement with the microbiological data.

## 4 DISCUSSION

Three preservation solutions were studied in this experiment, and it was found that all of them could inhibit the growth and proliferation of microorganisms and delay the onset of spoilage. The preservation solutions could reduce microbial counts by an average of 1–2 log CFU/g. However, the bacteria inhibitory effect gradually decreased during the storage. One reason for the phenomenon is attributed to the antimicrobial components such as nisin, polyphenol, and curcumin. in the preservation solution that were neutralized by the pork components or permeated inward rapidly after contacting the surface of the pork. Other reasons could be that the bacteria developed tolerance to the antimicrobial components with time during the bacteria growth, or the components degraded or degraded with time and lost their potency.

During low temperature storage, the main bacteria on chilled pork were Psychrophiles. The reason why they are adapted to low temperature is that the saturated fatty acids in their cytoplasm were transformed into unsaturated fatty acids during the evolutionary process (Nash & Grant, 1969). The unsaturated fatty acids can keep the fat of the cytoplasm membrane in a fluid state allowing membrane transportation of various substances, to ensure the survival, growth, and propagation of psychrophiles. Furthermore, the enzyme system of psychrophiles has also undergone evolutionary changes in structural and functional properties to enable high biocatalytic activity at low temperatures (Cavicchioli, Thomas, & Curmi, 2000).

The three preservation solutions exhibited different bacteria inhibitory effects on chilled pork. Solution A showed the best preservative ability. According to the experimental results of microbial analysis, TVB‐N, and sensory parameters, solution A is a good natural preservation solution. The numbers of total bacteria, *Pseudomonas*, *Enterobacteriaceae*, lactic acid bacteria, *B. thermosphacta*, and psychrophile were 5.18 ± 0.25 log CFU/g, 5.34 ± 0.30 log CFU/g, 5.00 ± 0.30 log CFU/g, 5.45 ± 0.25 log CFU/g, 1.65 ± 0.17 log CFU/g, and 3.98 ± 0.27 log CFU/g, respectively. TVB‐N value was 11 mg/100 g at day 21 during storage at 4 ± 1°C. Natural preservative cocktail A comprised of green tea polyphenol, chitosan, propolis, and nisin. As reported, catechins, found in green tea, have been shown to have antimicrobial effects against several bacterial pathogens (Nicola & David, 2010). Chitosan possesses high antibacterial activity, broad spectrum of activity, and low toxicity toward mammalian cells. Chitosan exhibits antibiofilm activities and the ability to damage biofilms formed by microbes, for instance *Candida albicans* (Torres‐Rêgo et al., 2019). Both the extract of spice and propolis showed antibacterial activity to *L. monocytogenes*, *E. coli*, *P. fluorescens*, and *L. sake*. (Zabaiou et al., 2017; Zhang, Wu, & Guo, 2016). As a natural green food preservative, nisin is the only bacteriocin approved by both U.S. FDA and the WHO so far (Zhang & Zhong, 2015). The combined effects of these components endowed solution A the most effective inhibitory activity against microbial growth. Therefore, preservation solution A is a good natural antimicrobial preservative solution.

Preservation solution B could also reduce the total microbe counts, as well as *Pseudomonas*, *Enterobacteriaceae*, and psychrophiles counts in chilled pork. However, compared with preservation solution A, solution B showed less inhibitory effect on the growth of lactic acid bacteria and *B. thermosphacta*. The TVB‐N value and biogenic amine of chilled pork treated with solution B decreased, and the chilled pork presented a bright red, which is useful in practical application in the meat industry. Hence, solution B could also be used for natural preservation on vacuum‐packaged chilled pork.

Solution C could inhibit the initial growth of *Pseudomonas* and *Enterobacteriaceae*, and decrease TVB‐N levels in the late stage of storage. However, the content of putrescine was relatively high in the late storage. Therefore, preservation C is not suitable for processing vacuum‐packaged chilled pork.

## 5 CONCLUSION

The results of this study showed that the shelf‐life of vacuum‐packed chilled pork was affected by using different natural preservatives cocktails at 4°C. The storage of vacuum‐packed chilled pork in three preservatives groups had a positive effect on the microbiological, biochemical, and sensory parameters compare to nontreated control meat. Though solution C could inhibit the initial growth of *Pseudomonas* and partially inhibited the growth of *Enterobacteriaceae*, the content of putrescine in the pork treated with solution C was as high as 30.14 ± 2.89 mg/kg after 21 days of storage at 4°C. Hence, Solution C is not an ideal preservative for vacuum‐packed chilled pork. Solution A showed the highest bacteria inhibitory effect and received the highest score of sensory evaluation among the three preservatives. Besides the microbial inhibitory activity, solution B endowed the meat appealing color. Thus, both solution A and solution B could be expected to become a good preservative to be applied in the meat industry for improving shelf‐life of vacuum‐packed chilled pork.

## CONFLICT OF INTEREST

The authors declare no conflict of interest, financial, or otherwise.

## ETHICAL APPROVAL AND CONSENT TO PARTICIPATE

The work reported in this manuscript did not involve use of human or animal subjects and, thus, did not require ethical approval and consent by the participating institutions.

## HUMAN AND ANIMAL RIGHTS

The study reported here did not require treatments for humans or animals; thus, human and animal rights concerns do not apply.

## CONSENT FOR PUBLICATION

The manuscript was verified and approved by all the coauthors listed for publication in “Current Pharmaceutical Biotechnology.”
